# Long-Term Clinical Outcomes of Ahmed Valve Implantation in Aniridic Glaucoma

**DOI:** 10.3390/biomedicines11112996

**Published:** 2023-11-08

**Authors:** Bartłomiej Bolek, Edward Wylęgała, Dorota Tarnawska

**Affiliations:** 1 Chair and Clinical Department of Ophthalmology, School of Medicine in Zabrze, Medical University of Silesia in Katowice, 40-760 Katowice, Poland; 2 Clinical Department of Ophthalmology, District Railway Hospital, 40-760 Katowice, Poland; 3 Institute of Biomedical Engineering, Faculty of Science and Technology, University of Silesia in Katowice, 75 Pułku Piechoty 1A, 41-500 Chorzów, Poland

**Keywords:** Ahmed valve, aniridia, glaucoma, glaucoma drainage device, intraocular pressure

## Abstract

Background: This study assessed the efficacy and safety of Ahmed valve implantation in patients with aniridic glaucoma for three consecutive years. Methods: Six adult patients (seven eyes) with Ahmed valve (AV) implants for aniridic glaucoma were enrolled in the study. The primary outcome measures were intraocular pressure reduction, glaucoma medication use, success rates, and visual acuity after AV implantation. A 30% reduction in IOP from baseline without the need for re-intervention was considered an effective treatment. The cessation of antiglaucoma medications was defined as complete success. Intraoperative and postoperative complications were included as secondary outcome measures. Measurements were performed preoperatively, at the first week, and 1, 3, 6, 12, 18, 24, 30, and 36 months postoperatively. Results: A total of seven eyes (6 patients) were evaluated 36 months after AV implantation. The mean ± SD values of IOP preoperatively at 1 day, 1 week, and 1, 3, 6, 12, 18, 24, 30, and 36 months postoperatively were 30.4 ± 4.0 mmHg, 14.6 ± 4.6 mmHg, 16.1 ± 4.6 mmHg, 20.7 ± 7.0 mmHg, 14.5 ± 2.7 mmHg, 16.5 ± 5.9 mmHg, 16.2 ± 4.0 mmHg, 16.3 ± 4.3 mmHg, 17.2 ± 10.1 mmHg, 17.6 ± 6.9 mmHg, and 18.2 ± 5.5 mmHg, respectively. At the last follow up, the mean IOP was reduced by 40.2%. The qualified success rate was 85.7%. One patient (one eye) at the last follow-up visit did not require antiglaucoma medications, resulting in a complete success rate of 14.3%. Intra- and postoperative mild or moderate subconjunctival bleeding was observed in all the patients. No other major/minor intraoperative or postoperative complications were noted. Conclusions: In long-term follow up, the AV implantation procedure is well-tolerated and relatively safe for reducing IOP in adult aniridia patients with glaucoma. These results should be validated through studies involving a larger patient cohort.

## 1. Introduction

Aniridia is a profoundly visually impairing rare genetic disorder primarily caused by a heterozygous mutation in paired box 6 (PAX6) [1,2]. This condition leads to the underdevelopment or abnormal development of the iris, and is associated with various ocular abnormalities, including keratopathy, cataract, nystagmus, foveal hypoplasia, optic nerve hypoplasia, and glaucoma. Secondary glaucoma usually develops during the first two decades of life with prevalence from 6% to 75% [3,4,5], or even higher [6]. Rudimentary iris stroma extends forwards onto the trabecular meshwork, at first resembling anterior synechiae, followed by forming a sheet that results in eventual angle closure [3]. Other mechanisms implicated in the pathogenesis of glaucoma involve the absence of the Schlemm canal [7]. Various surgical techniques, including goniosurgery, trabeculotomy, trabeculectomy, the use of glaucoma drainage devices (GDD), and cyclodestructive procedures, have been employed to address this type of glaucoma, yielding variable degrees of success [8,9,10,11,12,13,14,15,16].

The purpose of this study was to evaluate the long-term outcomes and complication rates of Ahmed valve (AV) implantation in patients with glaucoma secondary to congenital aniridia. The primary outcome measures encompassed reductions in intraocular pressure (IOP), changes in the usage of glaucoma medications, success rates, and alterations in visual acuity following the AV implantation. Additionally, secondary outcome measures involved the evaluation of both intraoperative and postoperative complications.

## 2. Materials and Methods

This study was a retrospective, single-arm case series, conducted in a noncomparative manner at a single center. The study received approval from the institutional review board of the Medical University of Silesia (Approval number: KNW/0022/KB1/131/16) and adhered to the principles of the Declaration of Helsinki. Patients who underwent AV implantation procedure (model FP7, New World Medical, Rancho Cucamonga, CA, USA) for aniridic glaucoma operated upon between November 2008 and April 2019 were included in the study. All patients provided informed consent for the AV implantation procedure. The study enrolled participants who met the following inclusion criteria: congenital aniridia, adult patients (≥18 years), AV implantation due to uncontrolled aniridic glaucoma (IOP > 21 mmHg, despite maximum tolerated doses of antiglaucoma medications), intolerance to glaucoma medications despite well-controlled IOP, 36-month follow-up period. Exclusion criteria encompassed the following: pregnant women, patients aged <18 years. Patients with a history of other treatments/surgeries for glaucoma were not excluded from the study.

A retrospective review of medical and surgical records was performed to collect the following data: complete ophthalmic examination with IOP measurements, number of antiglaucoma medications, and best-corrected log MAR visual acuity recorded preoperatively and at 1 day, 1 week, and 1, 3, 6, 12, 18, 24, 30, and 36 months after surgery.

All follow-up time points and IOP measurements in our clinic are always made according to the World Glaucoma Association Guidelines on Design and Reporting of Glaucoma Surgical Trials—the average of three measurements obtained with the standard Goldmann applanation tonometer (GAT) [17]. Data also extracted were age, sex, concomitant ocular disorders, history of other intraocular surgeries, lens status, intraoperative complications, postoperative complications, and re-interventions.

Treatment was deemed successful (qualified success) if, during 36-month follow-up assessments, there was a 30% reduction in IOP compared to the baseline value, and this reduction was achieved without requiring surgical re-intervention. Increased IOP was counted as a failure if it occurred at two consecutive follow-up visits. IOP spikes to 3 months after AV implantation were not considered as a failure. Complete success was defined as cessation of antiglaucoma medications.

In cases where IOP was not adequately reduced during follow up, additional ocular hypotensive medications were added. In the event of failure to achieve IOP control with up to three topical medications, the planned intervention in the study group was to be transscleral cyclophotocoagulation (TSCPC).

AV implantation procedure was performed under peribulbar or general anesthesia. All the plates were implanted at the superior temporal quadrant of the eye. First, a traction suture was placed into the superior limbal cornea. A 10 mm conjunctival and a fornix-based opening were created in the superior temporal quadrant. The plate was placed in sub-Tenon space 8–9 mm from the limbus between the lateral and superior rectus muscles and sutured to the superficial sclera with 8-0 Prolene nonabsorbable sutures. Next, the tube was trimmed to extend 2–3 mm into the anterior chamber. A 23-gauge needle was used to penetrate sclera 2–3 mm posterior to the corneal limbus, directed parallel to the iris surface. The tube was inserted bevel up through the tunnel into the anterior chamber and secured to the sclera using one 10-0 nylon anchoring suture. The silicone tube near the limbus was then covered with a 5 × 6 mm full-thickness donor sclera graft, sutured with four 7-0 absorbable (polyglactin) sutures. The conjunctiva was closed using interrupted 10-0 nylon sutures. A subconjunctival steroid and antibiotic injection were performed. All the procedures were performed by two experienced surgeons (D.T. and E.W.).

In the first week after surgery, anti-glaucoma medication was withdrawn in all but one patient. At subsequent follow-up visits, they were added as needed, depending on IOP.

Postoperatively, patients were treated topically with dexamethasone (five times a day for two weeks and tapered over two months) and ofloxacin (five times a day for two weeks). Statistical analysis utilized Statistica Software version 13 (TIBCO Software Inc., Palo Alto, CA, USA). Datasets were compared based on data distribution, with the Wilcoxon signed-rank test or paired t-test applied for relevant parameters. Kaplan–Meier survival curves assessed treatment success over time, and IOP results were visualized using a scatter plot comparing preoperative IOP (*x*-axis) with 3-year postoperative IOP (*y*-axis). A significance level of *p* ≤ 0.05 denoted statistical significance. 

## 3. Results

**Figure 1:**
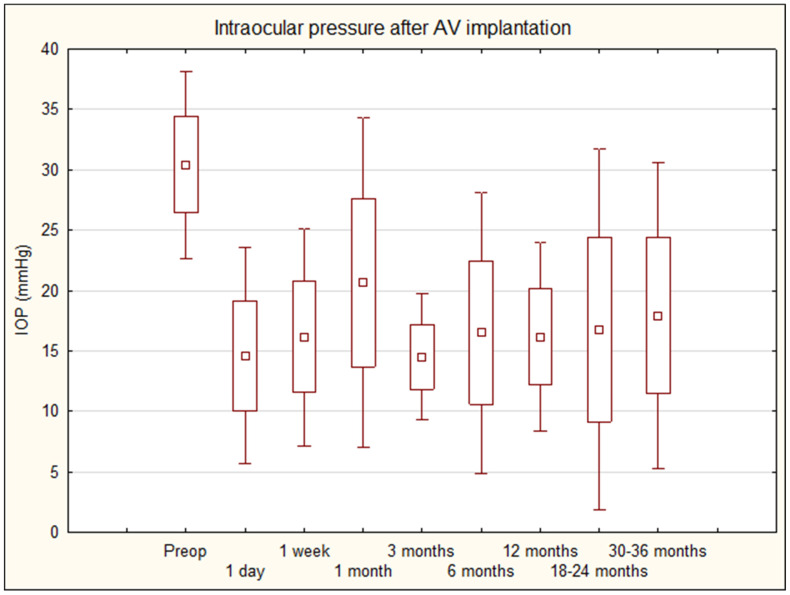
Intraocular pressure after AV implantation—36-month follow up. *AV*—*Ahmed valve*; *IOP*—*intraocular pressure*.

Six adult patients (seven eyes) with aniridic glaucoma were enrolled for AV implantation. Patient characteristics are detailed in Table 1. None of the patients required reoperation throughout the follow-up period due to failure to achieve the target IOP. In the data collected, there were three follow-up time-points not available due to patient missed visits (case n6—day 1 visit; case n4—18-month visit; case n1—30-month visit). The mean ± SD values of IOP preoperatively at 1 day, 1 week, and 1, 3, 6, 12, 18, 24, 30, and 36 months postoperatively were 30.4 ± 4.0 mmHg, 14.6 ± 4.6 mmHg, 16.1 ± 4.6 mmHg, 20.7 ± 7.0 mmHg, 14.5 ± 2.7 mmHg, 16.5 ± 5.9 mmHg, 16.2 ± 4.0 mmHg, 16.3 ± 4.3 mmHg, 17.2 ± 10.1 mmHg, 17.6 ± 6.9 mmHg, and 18.2 ± 5.5 mmHg, respectively (Table 1 and Table 2, Figure 1). The mean IOP at the final follow up exhibited a reduction of 40.2% (Table 2). The qualified success rate was 85,7% (six eyes). One patient (one eye) (at 30- and 36-month follow-up visit) did not achieve IOP reduction at two consecutive follow-up visits; this was considered a failure and qualified for TSCPC. However, the procedure was canceled because the patient had an intraocular pressure of 18 mmHg on the day of admission to the laser procedure. In further follow up, already beyond the three-year period of the study, the patient maintained an effectively lowered IOP, and at the 84-month visit 16 mm IOP was recorded with two antiglaucoma medications.

**Figure 2:**
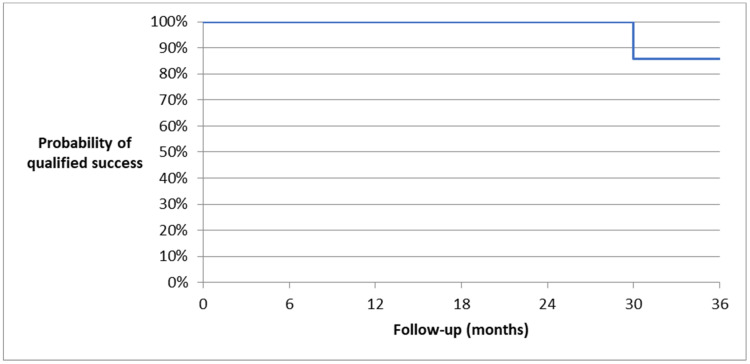
Kaplan–Meier survival curve of qualified success after AV implantation—36-month follow up.

**Figure 3:**
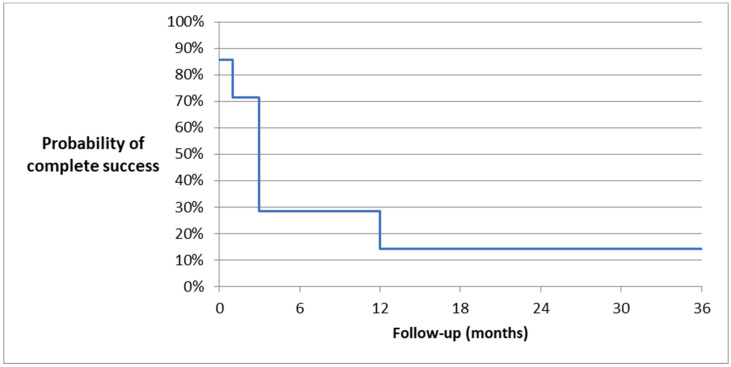
Kaplan–Meier survival curve of complete success after AV implantation—36-month follow up.

**Figure 4:**
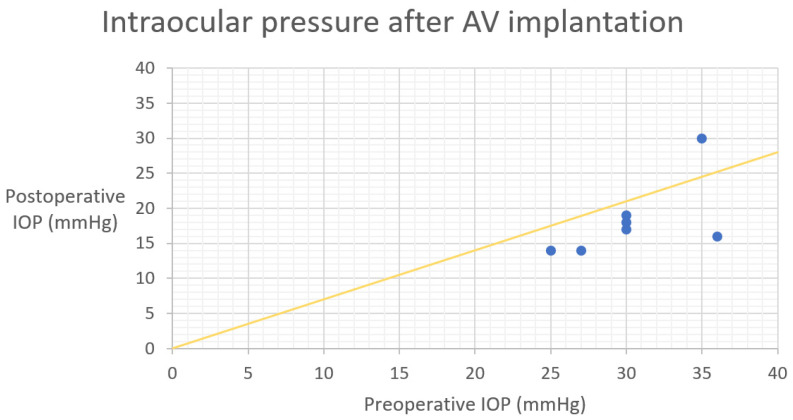
Scatter plot as preoperative IOP (*x*-axis) versus 3-year postoperative IOP (*y*-axis). Yellow slope diagonal line represents 30% IOP reduction. *AV*—*Ahmed valve*, *IOP*—*intraocular pressure*.

At the 36-month follow up, one patient (one eye) no longer needed antiglaucoma medications. The complete success rate was 14.3% (Figure 2 and Figure 3—Kaplan-Meier survival curves, Figure 4—scatter plot).

**Figure 5:**
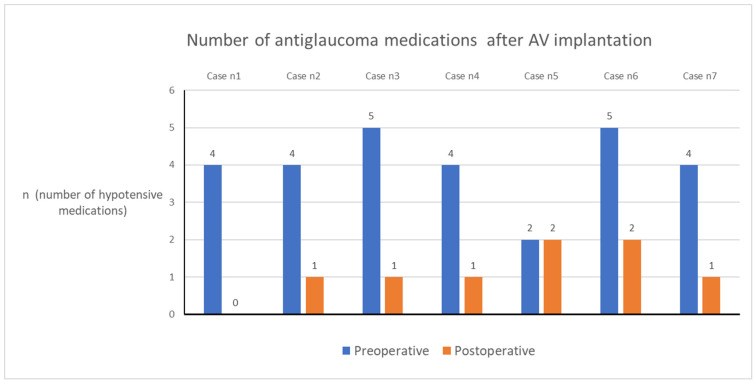
Number of antiglaucoma medications preoperative and postoperative at last follow up. *AV*—*Ahmed valve*.

The mean ± SD values of the number of antiglaucoma medications preoperatively and at 1 day, 1 week, and 1, 3, 6, 12, 18, 24, 30, and 36 months postoperatively were 4.0 ± 1.0, 0.2 ± 0.4, 0.1 ± 0.4, 0.3 ± 0.5, 0.8 ± 0.8, 0.8 ± 0.8, 1.3 ± 0.5, 1.5 ± 1.3, 1.8 ± 1.1, 1.4 ± 0.9, and 1.3 ± 0.6, respectively (Table 2, Figure 5). Before AV implantation, two patients required systemic carbonic anhydrase therapy. However, none of the patients needed systemic carbonic anhydrase treatment at the 36-month follow-up visit. Five patients had reduced the number of antiglaucoma medications they were taking, and two had maintained the same number of medications at the last follow-up visit compared to the baseline. Importantly, none of the patients required more antiglaucoma medications compared to their preoperative regimen at the last visit. None of the patients required systemic carbonic anhydrase during the 36-month follow up.

The mean best-corrected log MAR visual acuity preoperatively at 1 day, 1 week, and 1, 3, 6, 12, 18, 24, 30, and 36 months postoperatively were 1.64 ± 0.32, 1.72 ± 0.23, 1.64 ± 0.35, 1.33 ± 0.23, 1.53 ± 0.37, 1.57 ± 0.30, 1.58 ± 0.35, 1.90 ± 0.76, 1.56 ± 0.15, 1.52 ± 0.22, and 1.53 ± 0.25, respectively.

**Figure 6:**
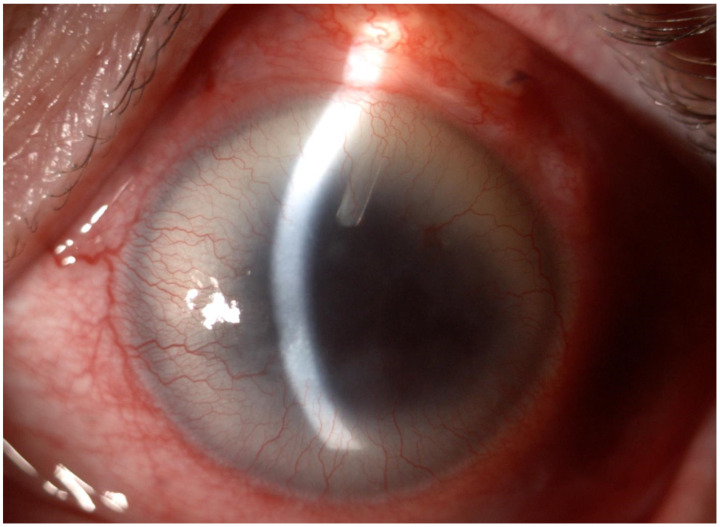
Left eye of the patient with aniridia and aphakia after Ahmed valve implantation. The valve tube in the anterior chamber is visible. Grade 2 aniridia-associated keratopathy can be noted.

Table 3 provides a comprehensive list of both intraoperative and postoperative complications. An IOP spike was observed in two patients (28.6%). Intra- and postoperative mild or moderate subconjunctival bleeding was observed in all the patients. No other major/minor intraoperative or postoperative complications occurred (Figure 6. Aniridia patient after AV implantation).

## 4. Discussion

Glaucoma secondary to aniridia is difficult to control with intraocular pressure-lowering medication. Frequently a surgical approach is needed, and few types of procedures are performed; however, the success rate is highly variable. Studies report the use of (1) aqueous drainage devices, (2) angle surgeries, especially before the angle closure is extensive, (3) filtering operations, and (4) cyclodestructive procedures.

Cyclocryotherapy and transscleral diode laser cyclophotocoagulation (TSCPC) are the two most common cyclodestructive methods described in the literature in aniridia-related glaucoma. The efficacy of the first method is mediocre/average, with a relatively high complication rate. Wallace and associates report that in six of nine procedures (performed in one or more sessions), IOP control was achieved; nonetheless, authors add that they would now prefer performing trabeculectomy or seton implantation before cyclocryotherapy [16]. Wiggins and associates evaluated different types of glaucoma surgeries in aniridia [7]. In the cyclocryotherapy group, 5 of 20 of the performed procedures were successful. The authors reported complications of this procedure, like phthisis bulbi and cataract. In their study, other glaucoma surgeries like trabeculotomy, trabeculectomy, and Molteno implant placement were performed, with a conclusion that the best results occurred after Molteno implants in aniridia patients, and authors advocate this method. Another study concerning cyclocryotherapy treatment reported that four (50%) of eight aniridic eyes had a “devastating complication developed, three of which represented phthisis and the fourth which represented a retinal detachment followed by phthisis” [18]. TSCPC is another type of cyclodestructive procedure which is used in patients with aniridia. The efficacy is similar to cyclocryotherapy; however, the complication rate is lower, with no literature reports of phthisis bulbi [14]. Three patients in our study had TSCPC before AV implantation. We did not observe any major complications postoperatively. However, due to worsening IOP control, these patients underwent AV implantation (an average of two years after TSCPC) and were enrolled in the study. The above-mentioned cyclodestrutive methods usually need to be repeated to achieve proper IOP control.

Therapeutic goniotomy is usually unsuccessful [10,19]. However, a few authors reported success of this procedure as a prophylactic before glaucoma develops [8,19]. Compared to goniotomy, better effectiveness in the treatment of aniridic glaucoma is achieved with trabeculotomy [10]. Adachi and associates reported that ten (83%) of twelve eyes obtained lOP control after the first or second trabeculotomy with a mean follow up of around ten years.

Trabeculectomy is a frequently performed procedure in aniridia, usually as a primary surgical approach. However, results in this type of glaucoma are unsatisfactory. In a previously cited paper, Wiggins and associates analyzed the efficacy of trabeculectomy with or without fluorouracil in aniridia patients [7]. The results were poor—out of fifteen procedures only one was successful. Similarly, in a different study, cited above, all five patients initially treated with trabeculectomy needed another glaucoma surgery in less than a one year follow up [9]. Durai and associates compared trabeculectomy to the Aurolab Aqueous Drainage Implant [11]. Again, the results of trabeculectomy were mediocre (the probability of failure at 2 years was 58.3%) and the efficacy of the Aurolab Aqueous Drainage Implant was definitely better (the probability of failure at two years was 11.1%). As mentioned in the description of patients’ characteristics, three of our patients had trabeculectomy before AV implantation. In these few cases, the efficacy of this procedure lasted longer, with an average of five years until AV implantation.

Few studies report the usage of different types of GDD in aniridic glaucoma. In a study mentioned earlier conducted by Wiggins and associates, out of five eyes with Molteno implants, only one required reoperation during a mean of 28 months’ follow up [7]. The same implant used by Molteno and associates allowed successful IOP control (defined as IOP < 20 mm Hg without medical treatment) in all three aniridia patients in a mean follow up of 5 years [13]. Billson and associates also reported reasonable IOP control with Molteno GDD over a three-year follow-up period in two patients [20]. In the study mentioned earlier conducted by Adachi and associates [9], the authors also implanted Molteno GDD with comparably good results—a success rate of 83% (five of six eyes had IOP < 21 mm Hg in the mean follow-up period of 10.4 years).

Only one study reported the effectiveness of treatment with Baerveldt GDD for aniridia, with a surgical success in 7 of 8 eyes over 11–39 months of follow up [21].

Two studies published so far have reported the results of AV implantation in aniridia-related glaucoma. Almousa and associates reported successful IOP control in seven of eight eyes over a mean follow up of 37.4 months [22]. In one eye, persistent vitreous hemorrhage and subsequent irreparable total retinal detachment occurred after the procedure. But, apart from this one, no other complication related to AV implantation, including tube exposure, were noted. In the second study, Demirok and associates implanted AV in six eyes, and the success rate was 66.6% at 12 months and 50.0% at the last follow up (range 12–36 months) [23]. In one patient, authors reported tube exposure at 1 month post operation and retinal detachment associated with vitreous hemorrhage. No other major complications were noted. In both studies presented above, there was no distinction between pediatric and adult patients.

The decrease in IOP and the number of antiglaucoma medications was significant at each follow-up time point in our study. The mean IOP at 36-month follow up was reduced by 40.2%. The surgical success rate was particularly high at 85.7%, which aligns with previously published results [22,23]. Furthermore, our study reported a low occurrence of major complications. We observed an IOP spike in two patients (28.6%) at one month postoperatively, which is common in GDD implantations. Nevertheless, at the following visit, IOP normalized. In all our patients, subconjunctival hemorrhage occurred both intraoperatively and postoperatively, which was related to the AV implantation procedure itself. We did not observe other major/minor intraoperative or postoperative complications, such as vitreoretinal hemorrhage, as reported in similar studies [22,23]. In our study group, none of the patients had tube exposure, which is one of the most threatening complications for the surgeon.

The current study has several limitations, including its retrospective, noncomparative design, the small number of cases, and the fact that it only involves adult patients. Nevertheless, it is the second largest clinical study and the most numerous adult study in the literature. Similar obstacles are common in aniridia studies due to the specificity of this condition.

Our results and the studies presented above reveal how challenging is to treat aniridic glaucoma. According to aniridia experts and a review of the literature, topical intraocular pressure lowering medications should be the first option in the treatment of aniridic glaucoma [24]. Prophylactic goniotomy could be considered when the rudimentary iris extends onto the trabecular meshwork and before glaucoma occurs. In cases of congenital or early-developing glaucoma, many clinicians recommend trabeculotomy. For more advanced stages of aniridic glaucoma, it appears that the GDD is the superior way to achieve that goal. Larger, randomized, and comparative studies are needed to determine the best surgical method for aniridia-related glaucoma.

## 5. Conclusions

The AV implantation procedure is relatively safe and well-tolerated for reducing IOP in adult aniridia patients with glaucoma in long-term follow up. These results should be validated through studies involving a larger patient cohort.

## Figures and Tables

**Table 1:** Patient characteristics and intraocular pressure outcome. IOP—intraocular pressure; LE—lens extraction; LE + IOL—lens extraction with intraocular lens implantation; PKP—penetrating keratoplasty; TSCPC—transscleral cyclophotocoagulation; Trab—trabeculectomy.

Case	Age (Years)	Gender	Prior Surgeries	Lens Status	Preoperative IOP (mmHg)	Postoperative IOP 36 Months (mmHg)
1	53	M	LE, TSCPC	Aphakic	30	18
2	52	M	LE	Aphakic	30	19
3	37	F	LE + IOL, TSCPC, Trab	Pseudophakic	36	16
4	40	F	LE + IOL, Trab	Pseudophakic	27	14
5	48	F	LE + IOL, PKP	Pseudophakic	35	30
6	50	F	LE + IOL, PKP, Trab	Pseudophakic	30	17
7	40	F	LE + IOL, Trab	Pseudophakic	25	14

**Table 2:** Intraocular pressure and number of hypotensive medications after AV implantation.

	Mean IOP ± SD			Number of Hypotensive Medications ± SD			% IOP Reduction
Preop	30.4	±	4.0	4.0	±	1.0	-
1 day	14.6	±	4.6	0.2	±	0.4	52.0
1 week	16.1	±	4.6	0.1	±	0.4	46.9
1 month	20.7	±	7.0	0.3	±	0.5	32.1
3 months	14.5	±	2.7	0.8	±	0.8	52.3
6 months	16.5	±	5.9	0.8	±	0.8	45.8
12 months	16.2	±	4.0	1.3	±	0.5	46.9
18 months	16.3	±	4.3	1.5	±	1.3	46.6
24 months	17.2	±	10.1	1.8	±	1.1	43.5
30 months	17.6	±	6.9	1.4	±	0.9	42.2
36 months	18.2	±	5.5	1.3	±	0.6	40.2

**Table 3:** Intraoperative and postoperative complications after AV implantation.

Complications	No.	%
**Intraoperative**		
Scleral/iris perforation	0/7	0
Anterior chamber shallowing	0/7	0
Subconjunctival hemorrhage	7/7	100
**Postoperative**		
*Early*		
Conjunctival hyperemia	3/7	42.9
Subconjunctival hemorrhage	7/7	100
Epithelial defects	2/7	28.6
IOP spike	3/7	42.9
Hypotony, choroid detachment	0/7	0
Hyphema	0/7	0
Uveitis	0/7	0
Vitreous hemorrhage	0/7	0
*Late*		
Tube exposure	0/7	0
Diplopia/strabismus	0/7	0
Hypotony, choroid detachment	0/7	0
Corneal decompensation	0/7	0
Retinal detachment	0/7	0
Phthisis bulbi	0/7	0

## Data Availability

The datasets generated during and/or analyzed during the current study are available from the corresponding author on reasonable request.

## Abbreviations

AV	Ahmed valve
IOP	Intraocular pressure
TSCPC	Diode laser transscleral cyclophotocoagulation
GDD	Glaucoma drainage device
IOP	Intraocular pressure
GAT	Goldmann applanation tonometer
SD	Standard Deviation
